# Impact of Long-Term Forest Enrichment Planting on the Biological Status of Soil in a Deforested Dipterocarp Forest in Perak, Malaysia 

**DOI:** 10.1100/2012/641346

**Published:** 2012-04-19

**Authors:** D. S. Karam, A. Arifin, O. Radziah, J. Shamshuddin, N. M. Majid, A. H. Hazandy, I. Zahari, A. H. Nor Halizah, T. X. Rui

**Affiliations:** ^1^Department of Forest Production, Faculty of Forestry, Universiti Putra Malaysia, 43400 Serdang, Selangor, Malaysia; ^2^Laboratory of Sustainable Bioresource Management, Institute of Tropical Forestry and Forest Products, Universiti Putra Malaysia, 43400 Serdang, Selangor, Malaysia; ^3^Department of Land Management, Faculty of Agriculture, Universiti Putra Malaysia, 43400 Serdang, Selangor, Malaysia; ^4^Laboratory of Food Crops and Floriculture, Institute of Tropical Agriculture, Universiti Putra Malaysia, 43400 Serdang, Selangor, Malaysia; ^5^Forestry Department Peninsular Malaysia, Jalan Sultan Salahuddin, 50660 Kuala Lumpur, Malaysia

## Abstract

Deforestation leads to the deterioration of soil fertility which occurs rapidly under tropical climates. Forest rehabilitation is one of the approaches to restore soil fertility and increase the productivity of degraded areas. The objective of this study was to evaluate and compare soil biological properties under enrichment planting and secondary forests at Tapah Hill Forest Reserve, Perak after 42 years of planting. Both areas were excessively logged in the 1950s and left idle without any appropriate forest management until 1968 when rehabilitation program was initiated. Six subplots (20 m × 20 m) were established within each enrichment planting (F1) and secondary forest (F2) plots, after which soil was sampled at depths of 0–15 cm (topsoil) and 15–30 cm (subsoil). Results showed that total mean microbial enzymatic activity, as well as biomass C and N content, was significantly higher in F1 compared to F2. The results, despite sample variability, suggest that the rehabilitation program improves the soil biological activities where high rate of soil organic matter, organic C, N, suitable soil acidity range, and abundance of forest litter is believed to be the predisposing factor promoting higher population of microbial in F1 as compared to F2. In conclusion total microbial enzymatic activity, biomass C and biomass N evaluation were higher in enrichment planting plot compared to secondary forest. After 42 years of planting, rehabilitation or enrichment planting helps to restore the productivity of planted forest in terms of biological parameters.

## 1. Introduction

Malaysia is a country rich in biodiversity of which natural forest is a home for thousands of flora and fauna [1]. However, the need for development and urbanization catalysed by the pressure of rising human population has made vast area of natural forests cleared up to cultivate new area for housing and wood productions. Liebig et al. [2] stated that the fertility of soil proportionally change with time catalyzed by natural phenomena and human activities. Hence, deforestation of natural forest leads to soil degradation, which proceeds rapidly under tropical climatic conditions [3, 4]. Forest rehabilitation is believed to be one of the best ways to overcome and lower down the demand for woody and nonwoody products from natural forest. Besides that, forest plantation also supports the shortage of wood supply, while sustaining world ecosystem [3]. In addition, forest plantation is also known as an alternative way to restore degraded sites to its original condition and sustains its soil fertility [5, 6]. Insam [7] found that soil fertility and its management are the most crucial part to evaluate a particular site of soil ecological area which gives a preview of the site's environmental management and the extent of success for a particular forest rehabilitation program which can only be identified through its soil fertility evaluation.

Enrichment planting is one of important technique used in forest rehabilitation [8, 9]. Montagnini et al. [10] defined enrichment planting as the introduction of valuable species to degraded forests without the elimination of valuable individual which already existed at that particular site. Adjers et al. [11] summarized that there are total of 25857 ha of forest plantation had been planted through enrichment planting technique in Peninsular Malaysia. *Shorea acuminata*, *S. leprosula*, *Dryobalanops aromatica*, and *D. oblongifolia* are among the favorite species planted in Peninsular Malaysia [12]. While for secondary forest, it is a forest area which has regrown trees after major disruption and disturbance such as fire and deforestation. Normally, the regeneration of plants species in secondary forests are done naturally by itself without any forest treatment given for a period of long time till the effect of disturbance is no longer noticed.

It is undeniable that soil microorganism is the major agents in promoting nutrient cycling including carbon (C), nitrogen (N), phosphorus (P), and sulphur (S). Furthermore, Gaspar et al. [13] concluded that soil microbial biomass comprises 1–4% and 2–6% of total organic C and N in soil, respectively. Rapid turnover of microbial activities in soil is dependent on the changes occurring in the surrounding environment such as climate change, disturbance, and pollutant toxicity [14, 15] which made microbial activity a good sensitive indicator [16] for soil fertility evaluation. Islam and Weil [17] also stressed the importance of including microbial biomass evaluation to describe the status of fertility and quality of soil at a particular study site.

Enzymatic activities are also one of the important evaluation aspects for determining soil fertility. They play a vital role in the organic residues degradation, humic substance synthesis, pollutant degradation, and nutrient cycles in soil [18]. Fluorescein diacetate (FDA) hydrolysis assay provides a reliable estimation of overall microbial activity in soil [19] and is widely used to analyse bacterial and fungal enzymatic activities [20, 21]. In addition, FDA analysis is considered as nonspecific because it is hydrolysed by various types of enzymes which include protease, esterase, and lipase [13, 21]. Heal and Maclean [22] found that approximately 90% of the energy transfer cycle in the soil was via microbial decomposer, and total microbial activity illustrates a general measurement of the organic matter turnover. Behera and Sahani [5] stated the importance of including biological studies, such as the evaluation of microbial biomass in land evaluations, because they provide a better indication of changes or degradation in forest soils than carbon and nitrogen analyses. Vásquez-Murrieta et al. [23] also stated that the key factors regulating and maintaining continuous supplies of nutrients in the soil for plant uptake are circulated by soil microbes. Soil fertility evaluation primarily focuses on the physicochemical properties in order to describe the growth performance of particular tree species at the plantation without taking into account the importance of soil biological properties as sensitive indicator to the changes occurring in the soil [24]. Hence, the objective of this study was to provide information and compare soil biological properties under enrichment planting and secondary forests after 42 years (as for 2010) of planting at Bukit Tapah Forest Reserve, Perak, Malaysia.

## 2. Materials and Methods

### 2.1. Description of the Study Site

**Figure 1:**
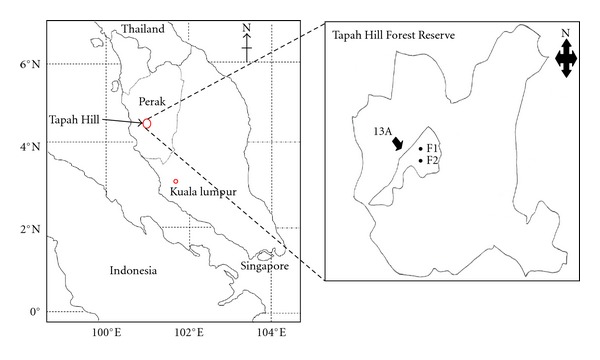
Enrichment planting (F1) and secondary forest (F2) plots at Tapah Hill Forest Reserve, Perak, Malaysia (Scale 1 : 20 000).

The study was carried out in enrichment planting (N 04.179394° E 101.31998°) and secondary forest (N 04.17336° E 101.31974°) at Bukit Tapah Forest Reserves, Perak (Figure 1) on 21st until 23rd July 2011. The mean annual rainfall and temperature are 2,417 mm and 24.5°C, respectively. The soils in this study area are classified as Ultisols, which are considered as highly weathered due to large amount of low-activity clays associated with high Al saturation [3]. All of the tree species of *Shorea leprosula*, *S. bracteolata*, and *S. macroptera* planted were done on 2nd February 1968, and the age of the trees was 42 years old in 2010, while adjacent secondary forest was left idle to undergo natural regeneration without any reforestation activity. Compartment 13 of Bukit Tapah is one of the 10 compartments that was gazetted for enrichment planting at Perak South District, Malaysia. About 1,185 hectares out of 64,984 hectares of Bukit Tapah Forest Reserve were converted to enrichment planting program of which compartment 13 covers 87.2 hectares of the forest reserves. The purpose of enrichment planting done at this area is to replace and curtail this particular area which had undergone excessive logging before 1968.

The size of the poly bags used to plant the seedlings was 10 cm × 15 cm × 23 cm. Twenty-six thousand five hundred and forty-four saplings were planted with 304 saplings per hectare, and the rates of survival recorded in 1970 found that only 9,158 trees managed to grow well and survive with resulting in 105 saplings per hectare, respectively. *Shorea leprosula*, *S. parvifolia*, *S. bracteolata*, and *S. macroptera *were the main species of Dipterocarpaceae planted in compartment 13 enrichment planting plot. The trees were planted on a 10 m × 3 m grid.

### 2.2. Experimental Design and Soil Sampling

This study used a completely randomized design. Enrichment planting and secondary forest plots were designated as F1 and F2, respectively. Six subplots were demarcated in each plot in order to serve as replicates. Six soil samples were randomly collected at depths of 0–15 cm and 15–30 cm in each subplot. The samples were then mixed together to form a composite sample for each soil depth range. Hence, 12 composite samples (six from soil depth 0–15 cm and six from soil depth 15–30 cm) were collected from each plot for the analysis. The composite samples were kept in UV-sterilized polyethylene bags at 0°–4°C.

### 2.3. Total Microbial Population

Spread-plate technique or direct count of colony forming unit was used to evaluate the estimation of microbial population [25, 26]. Nutrient agar was used for bacterial culture. Dilution factor of 10^−2^, 10^−3^, and 10^−4^ was found to be suitable for colony calculation after few pilot test carried out to standardize the dilution factor for every population counts. The number of colony forming units per gram soil was calculated using the following equation:

(1) $$\begin{array}{c} \text{number}\;\text{of}\;\text{colony}\;\text{forming}\;\text{units}/\text{g}\;\text{of}\;\text{dry}\;\text{weight}\;\text{soil}\\ =\frac{\left[\left(\text{mean}\;\text{plate}\;\text{count}\right)\left(\text{dilution}\;\text{factor}\right)\right]}{\left(\text{dry}\;\text{weight}\;\text{soil},\;\text{initial}\;\text{dilution}\right)}, \end{array}$$

where dry weight soil = (Weight of moist soil, initial dilution blank) × [(1 − % moisture soil sample)/100]. The results were expressed in log_10_ g^−1^ soil.

### 2.4. Microbial Enzymatic Activity

Fluorescein diacetate (FDA) hydrolysis assay illustrated by Sánchez-Monedero et al. [18] and Gagnon et al. [27] was used to evaluate microbial enzymatic activity.

### 2.5. Microbial Biomass Analysis

Soil microbial biomass C (MBC) and N (MBN) were extracted using rapid chloroform fumigation extraction described by Witt et al. [28]. Soil MBC analysed by wet dichromate oxidation [23] and calculation for biomass C is as below:

(2) $$\text{MBC}=\frac{\left({\text{C}}_{\text{fumigated}}-{\text{C}}_{\text{control}}\right)}{\text{kEC}}.$$

The chloroform-labile C pool was calculated as the difference between samples of un-fumigated and fumigated C which is proportional to MBC, where kEC is soil specifically estimated as 0.38 [29].

Soil MBN was determined using Kjeldahl digestion and distillation technique [30, 31]. The calculation for biomass N is

(3) $$\text{MBN}=\frac{\left({\text{N}}_{\text{fumigated}}-{\text{N}}_{\text{control}}\right)}{\text{kEN}}.$$

The chloroform-labile N pool was calculated as the difference between samples of un-fumigated and fumigated N which is proportional to MBN, where kEN is soil specifically estimated as 0.54 [32].

### 2.6. Measurement of Soil Organic Matter, Organic C, Total N, Soil Acidity, Bulk Density, and Moisture Content

Soil organic matter and organic C were determined using loss on ignition method [33] total N via Kjeldahl digestion [31], and soil acidity was elucidated in a 1 : 2.5 of soil : distilled water suspension using a glass electrode [34, 35]. Bulk density was determined using the disturbed soil technique, and the gravimetric method was used to measure soil moisture content.

## 3. Statistical Analysis

Student's *t*-test was used to compare the differences between the mean values for microbial population, enzymatic activity, biomass C, biomass N, and selected physicochemical properties for samples collected at the same depths in the adjacent plots. Pearson correlation analysis was used to detect the correlation between microbial biomass C with organic matter and microbial biomass N with total N. SPSS version 16.0 was used for the statistical analysis.

## 4. Results

**Figure 2:**
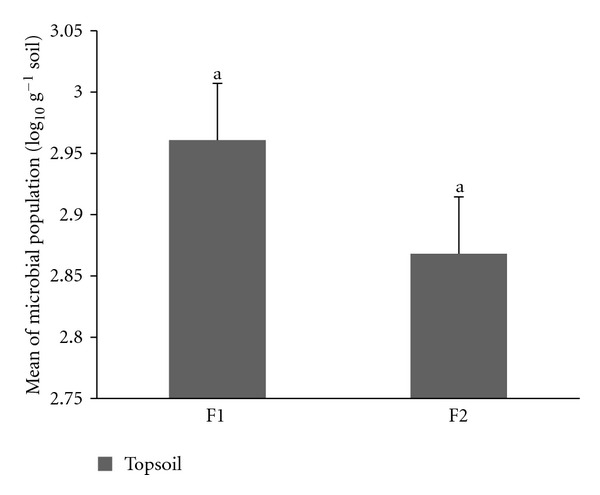
Total mean microbial population at F1 and F2 plots. Different letters indicate significant difference between means of the same soil depths at enrichment planting (F1) compared to secondary forest (F2) plots, using the Student's *t*-test (*P* ≤ 0.05) (bars are means, whiskers indicate standard error).

There were no significant differences (*P* ≤ 0.05) between F1 (2.96 ± 0.04 log_10_ g^−1^ soil) and F2 (2.87 ± 0.06 log_10_ g^−1^ soil) for microbial population count (Figure 2). The total mean of microbial population count for 15–30 cm depth for both plots was too low and was excluded from the final results to avoid bias.

**Figure 3:**
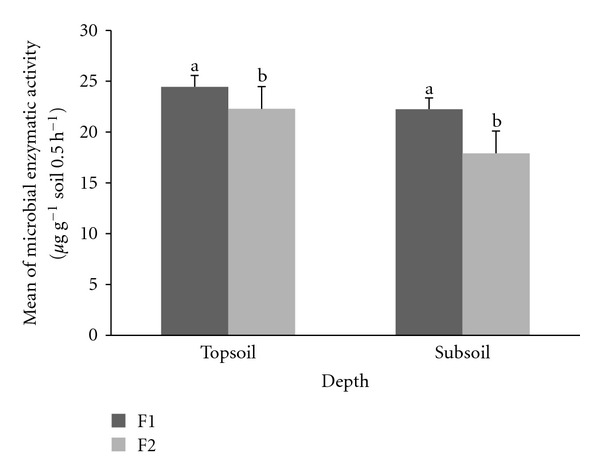
Total mean microbial enzymatic activity at F1 and F2 plots. Different letters indicate significant difference between means of the same soil depths at enrichment planting (F1) compared to secondary forest (F2) plots, using the Student's *t*-test (*P* ≤ 0.05) (bars are means, whiskers indicate standard error).

Microbial enzymatic activities were significantly different (*P* ≤ 0.05) for both F1 and F2 at each depth (Figure 3). Total mean of microbial enzymatic activity rate was 24.45 ± 0.65 *μ*g g^−1^ soil 0.5 h^−1^ and 22.91 ± 0.53 *μ*g g^−1^ soil 0.5 h^−1^ under F1 and F2 at 0–15 cm depths while, for 15–30 cm depth, F1 and F2 enzymatic activity rate each 22.25 ± 0.49 *μ*g g^−1^ soil 0.5 h^−1^ and 17.91 ± 1.73 *μ*g g^−1^ soil 0.5 h^−1^, respectively.

**Figure 4:**
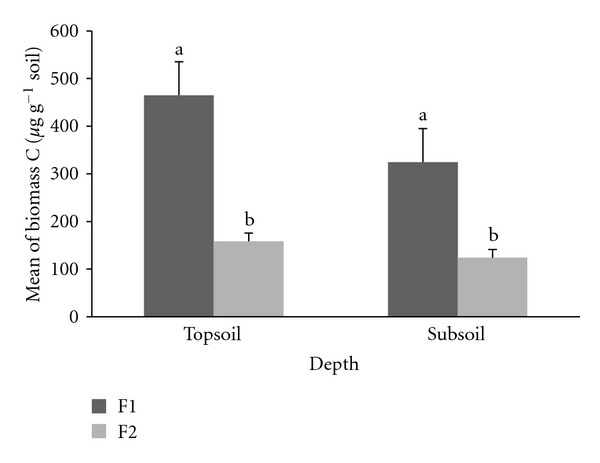
Total mean microbial biomass C at F1 and F2 plots. Different letters indicate significant difference between means of the same soil depths at enrichment planting (F1) compared to secondary forest (F2) plots, using the Student's *t*-test (*P* ≤ 0.05) (bars are means, whiskers indicate standard error).

MBC rate was significantly higher (*P* ≤ 0.05) in F1 compared to F2 at the same soil depths (Figure 4). Total mean of MBC rate for each F1 and F2 at 0–15 cm and 15–30 cm depths was 465 ± 105 *μ*g g^−1^ soil, 325 ± 58 *μ*g g^−1^ soil, 158 ± 66 *μ*g g^−1^ soil, and 124 ± 35 *μ*g g^−1^ soil, respectively.

**Figure 5:**
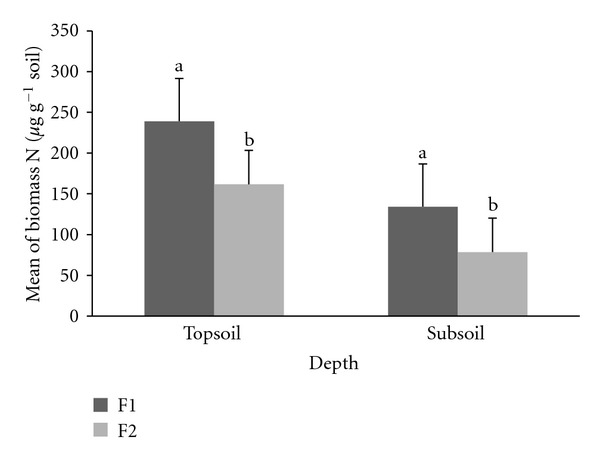
Total mean microbial biomass N at F1 and F2 plots. Different letters indicate significant difference between means of the same soil depths at enrichment planting (F1) compared to secondary forest (F2) plots, using the Student's *t*-test (*P* ≤ 0.05) (bars are means, whiskers indicate standard error).

**Figure 6:**
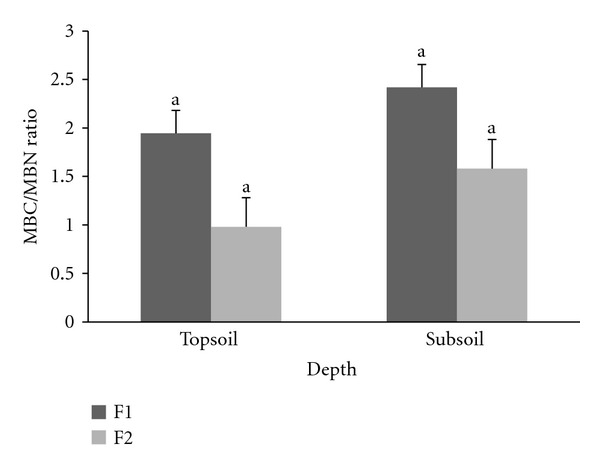
Ratio of microbial biomass C to microbial biomass N (MBC/MBN) between the same soil depths at enrichment planting (F1) and secondary forest (F2) plots. Different letters indicate significant difference between means of the same soil depths comparing enrichment planting (F1) to secondary forest (F2) plots, using the Student's *t*-test (*P* ≤ 0.05) (bars are means, whiskers indicate standard error).

F1 and F2 were significantly different (*P* ≤ 0.05) where F1 contained higher rate of MBN compared to F2 at 0–15 cm and 15–30 cm soil depths (Figure 5). Total mean of MBN rate for each F1 and F2 plots was 239 ± 8 *μ*g g^−1^ soil and 162 ± 18 *μ*g g^−1^ soil at 0–15 cm depth and 134 ± 12 *μ*g g^−1^ soil and 78 ± 11 *μ*g g^−1^ soil at 15–30 cm depth, respectively. There were no significant differences of ratio of MBC/MBN between F1 and F2 plots (*P* ≤ 0.05) (Figure 6). MBC/MBN ratio for F1 at 0–15 cm and 15–30 cm depths was 1.91 ± 0.41 and 2.48 ± 0.48. In contrast, F2 exhibits a lower MBC/MBN ratio of 1.03 ± 0.45 at 0–15 cm depth and 1.84 ± 0.49 at 15–30 cm depth.

Soil organic matter and organic C were significantly different (*P* ≤ 0.05) for both F1 and F2 at 0–15 cm and 15–30 cm depths (Table 1). Soil acidity does not show any significant difference for both plots at the same soil depths. At 0–15 cm, there were significant differences in bulk density and moisture content compared to F2. However, there were no significant differences detected between F1 and F2 at 15–30 cm.

Table 2 shows the results of the Pearson correlation for selected chemical and biological properties in both plots. There were no linear relationship detected between microbial biomass C and organic matter for both plots at the same soil depths. Besides that, microbial biomass N and total N also do not show any linear relationship between the same soil depths. Correlation analysis of organic matter content and MBC/MBN ratios showed no strong relationship.

## 5. Discussion

Microbial population count between enrichment planting and secondary forest show a proportional in microbial growth, and this situation could be catalysed by the abundance of forest litter available on the forest floor which promotes microbial decomposing activity to take place and increase soil fertility [5, 36].

Microbial enzymatic activity was found to be higher in 0–15 cm depth compared to the lower depth, and also greater in enrichment planting compared to secondary forest. This activity is probably facilitated by the thicker and greater abundance of forest litter available, which enhances microbial decomposing processes. Higher content of organic matter in enrichment planting as compared with secondary forest contributes to the higher enzymatic activity. Smith and Paul [16] justified that microbial activity has been proven to be a “sensitive indicator” to illustrate changes in soil organic matter. The higher microbial activity of the enrichment plot at 0–15 cm may also be due to the high moisture content, which, along with surrounding humidity, enhances the microorganism cycles in the soil. Moreover, low soil compaction in the enrichment plot would also provide better air and water penetration in the soil to allow macro- and microorganisms to thrive and undergo necessary daily biochemical processes.

Greater amount of organic matter in enrichment planting is a valuable indication of greater amount of MBC. Islam and Weil [17] suggested that abundance and thickness of the layer of litter on the forest floor promotes high decomposing processes by soil microorganism. In addition, Powlson et al. [37] claimed the sensitivity posed by labile C is proportional to the limitation of soil microbial biomass, and this affects organic C aggradation.

MBN in enrichment planting is greater compared to secondary forest for both soil depths. Higher MBN could be due to the higher total N availability possessed by enrichment planting compared to secondary forest. Kandeler et al. [38] observed that increase in microbial N might be reflected by the competition between microorganism and plants in limited N ecosystem condition. Hence, these results proved that changes in N whether it increases or decreases will catalyze the level of MBN as what we can observe at enrichment planting and secondary forest, respectively.

Variation of MBC/MBN ratio between enrichment planting and secondary forest shows the qualitative changes occurring in the soil biological composition [5]. The ratio of MBC and MBN was found to be proportional due to the same gradient level for both enrichment planting and secondary forest and [39] explained that reasonably high soil organic substrate and low total N compared to organic C at both sites are believed to be similar. Arifin et al. [3] and Carter [40] evaluated that the restoration of soil organic substrate in soil also depends on the carrying capacity, solum type, climate, and land usage management of soil. Likewise, the vast diversity of the organic substrate production in enrichment planting which promotes and sustains the food chain in soil contributes to sustaining an ideal amount of microbial biomass per unit soil [39].

The high acidity at both plots could be due to the formation of decomposition byproducts such as humic and fulvic acids [5], which decrease soil pH. At both forests, abundance of forest litters provides suitable medium for soil macro- and microdecomposer to break down forest litter constituent to release macro- and micronutrients to the soil to increase the soil fertility. However, soil microorganisms in tropical dry environment are found to be able to withstand high acidic condition in the soil as long as the pH does not decrease to the point where H^+^ ions begin to form precipitation products [41].

## 6. Conclusion

Total microbial enzymatic activity, biomass C, and N were found to be higher in enrichment planting plots compared to secondary forest. The abundance of organic substrate and increased soil acidity play important roles in the biological properties at both sites. The soil biological properties in enrichment planting were found to be improved compared to secondary forest after 42 years of planting. It is recommended that further research be done to determine the most sensitive microorganisms that caused the changes in the soil. Biological components of soils help in increasing the fertility of the soils by enhancing the retention capacity of nutrients for plant uptake and, thus, promoting the soil fertility and productive capability especially in the tropical environment condition. Further research must be conducted to identify the microorganisms that are most influential in soil changes. Biological properties of soil help increase fertility by enhancing the retention of nutrients available for plant uptake, thus, promoting soil fertility and productivity, especially in tropical environments. In conclusion, 42 years of forest enrichment planting using indigenous dipterocarp species led to recovery or restoration of soil biological properties to levels higher than observed in secondary forest. Therefore, forest enrichment planting by the Forestry Department Peninsular Malaysia effectively increased the productivity and fertility of soil in previously degraded forestland.

## Figures and Tables

**Table 1:** Selected soil physicochemical properties of enrichment planting (F1) and secondary forest (F2) plots.

Parameters	F1	F2	*P* value
	0–15 cm depth		

Organic matter (%)	16.99 ± 0.84^a^	12.12 ± 0.35^b^	0.001947
Organic carbon (%)	9.86 ± 0.49^a^	7.03 ± 0.47^b^	0.001947
Total nitrogen (%)	1.55 ± 0.09^a^	1.11 ± 0.09^b^	0.006318
pH-H_2_O	4.36 ± 0.11^a^	4.19 ± 0.05^a^	0.348473
Bulk density (g cm^−3^)	1.16 ± 0.01^a^	1.24 ± 0.02^b^	0.007088
Moisture content (%)	26.33 ± 0.61^a^	20.50 ± 1.91^b^	0.015656

	15–30 cm depth		

Organic matter (%)	14.29 ± 0.35^a^	11.27 ± 0.78^b^	0.005467
Organic carbon (%)	8.29 ± 0.20^a^	6.54 ± 0.45^b^	0.005466
Total nitrogen (%)	0.81 ± 0.05^a^	0.77 ± 0.10^a^	0.713792
pH-H_2_O	4.42 ± 0.10^a^	4.23 ± 0.08^b^	0.059146
Bulk density (g cm^−3^)	1.22 ± 0.01^a^	1.26 ± 0.02^a^	0.153677
Moisture content (%)	23.33 ± 0.49^a^	19.17 ± 2.60^a^	0.146512

Note: different letters each row indicate significant differences between the means of soil properties at both depths at enrichment planting (F1) or secondary forest (F2) plots using the Student's *t*-test (*P* < 0.05).

**Table 2:** Pearson correlation analysis results comparing microbial biomass C (MBC) with organic matter (OM), microbial biomass N (MBN) with total N (TN), and OM with MBC/MBN ratio for both plots at the same soil depths.

	MBC versus OM		MBN versus TN		OM versus MBC/MBN ratio	
Soil depth (cm)	*P* value	*r* ^2^	*P* value	*r* ^2^	*P* value	*r* ^2^
F1 (0–15)	0.197	0.708	0.087	0.749	0.830	0.113
F1 (15–30)	0.091	0.864	0.603	−0.271	0.667	−0.226
F2 (0–15)	0.947	−0.036	0.120	0.702	0.202	−0.606
F2 (15–30)	0.215	−0.593	0.939	0.040	0.146	−0.670

Note: F1: enrichment planting; F2: secondary forest.
